# Student feedback on the use of paintings in Sparshanam, the Medical Humanities module at KIST Medical College, Nepal

**DOI:** 10.1186/1472-6920-11-9

**Published:** 2011-03-08

**Authors:** P Ravi Shankar, Rano M Piryani, Kshitiz Upadhyay-Dhungel

**Affiliations:** 1 Dept. of Medical Education, KIST Medical College, Lalitpur, Nepal; 2 Dept. of Medicine, KIST Medical College, Lalitpur, Nepal; 3 Dept. of Physiology, KIST Medical College, Lalitpur, Nepal

## Abstract

**Background:**

Paintings have been used in Medical Humanities modules in Nepal at Manipal College of Medical Sciences and KIST Medical College. Detailed participant feedback about the paintings used, the activities carried out, problems with using paintings and the role of paintings in future modules has not been previously done. Hence the present study was carried out.

**Methods:**

The present module for first year medical students was conducted from February to August 2010 at KIST Medical College, Nepal. Paintings used were by Western artists and obtained from the Literature, Arts and Medicine database. The activities undertaken by the students include answering the questions 'What do you see' and 'What do you feel' about the painting, creating a story of 100 words about the scene depicted, and interpreting the painting using role plays and poems/songs. Feedback was not obtained about the last two activities. In August 2010 we obtained detailed feedback about the paintings used.

**Results:**

Seventy-eight of the 100 students (78%) participated. Thirty-four students (43.6%) were male. The most common overall comments about the use of paintings were "they helped me feel what I saw" (12 respondents), "enjoyed the sessions" (12 respondents), "some paintings were hard to interpret" (10 respondents) and "were in tune with module objectives" (10 respondents). Forty-eight (61.5%) felt the use of western paintings was appropriate. Suggestions to make annotations about paintings more useful were to make them shorter and more precise, simplify the language and properly introduce the artist. Forty-one students (52.6%) had difficulty with the exercise 'what do you feel'. Seventy-four students (94.9%) wanted paintings from Nepal to be included.

**Conclusions:**

Participant response was positive and they were satisfied with use of paintings in the module. Use of more paintings from Nepal and South Asia can be considered. Further studies may be required to understand whether use of paintings succeeded in fulfilling module objectives.

## Background

Medical humanities (MH) has been defined as 'an interdisciplinary, and increasingly international endeavor that draws on the creative and intellectual strengths of diverse disciplines, including literature, art, creative writing, drama, film, music, philosophy, ethical decision making, anthropology and history in pursuit of medical educational goals'[1]. MH programs are common in the West but are not so in South Asia.

### Art in medical education

Art has been used in medical education in the west. At Yale University School of Medicine in the United States (US) fine art was used to enhance the visual diagnostic skills of first year medical students [2]. In a family practice residency program in the US, visual thinking strategies (VTS) were used with the help of art images. Facilitated discussion about the art image was used to help people observe carefully, express their observations and ideas and build on the thoughts of others [3]. Paintings and videos have been used to teach professionalism to residents in a US medical school [4].

A common reason stated for incorporating art in medical education is that art may act as a balance to the dominance of medical science [5]. The aim is not only developing skills, but also facilitating personal growth and professional development. At the University of Missouri-Kansas City School of Medicine in the US 'The body image in medicine and the arts' course uses literature, photography, art, anthropology, art history, cultural studies, feminism, modernism, and medicine among other modalities to encourage students to interpret the human body more broadly [6]. A voluntary module was conducted at Manipal College of Medical Sciences (MCOMS), Pokhara, Nepal for interested students and faculty members in 2007 [7]. Participant feedback about the module was positive.

### Visual arts and clinical skills

One paper suggests that the visual arts can be used to help medical students increase their observational and interpretive skills [8]. In 2001 University of Cincinnati initiated a program called the 'Art of observation' (AOO) as an elective for second year students. The program used guided instruction in observation, description, interpretation and reflection of the visual arts [9]. AOO positively influenced clinical skills and also led to a sense of personal development as a physician. A 2001 survey among US Medical schools indicated that over half the schools incorporate art in their learning activities [10]. At the University of California at Irvine, year 3 medical students were divided into two groups [11]. The first small group participated in training using clinical photographs and paper cases while the second group used art and dance. Training using clinical material developed pattern recognition skills while training using art developed skills of emotional recognition, increased empathy, improved story and narrative and helped them identify multiple perspectives.

### Sparshanam, the Medical Humanities module

At KIST Medical College a MH module titled Sparshanam ('touch' in Sanskrit) is being conducted for first year medical students [12]. We wanted to emphasize the importance of touch and human contact in the doctor-patient relationship. Case scenarios, role-plays, paintings, debates, presentations and other activities were used to explore MH. Touch may not be directly connected to paintings and we had not used patients in the module but the module is a part of early clinical exposure during which students interact with patients for history taking during the first year. In a previous article we had briefly described the use of paintings in the MH module [13].

Our module 'Sparshanam' explores Medical Humanities through the power of human relationships. Among the specific objectives of the module were to understand empathy and its importance in medical care, appreciate the patient perspective of sickness and health, know the effect of disease and sickness of a loved one on the family, be aware of the doctor-patient relationship and recent developments in this vital area and be familiar with what it means to be sick in Nepal. Paintings play an important role in the module and their use has been described in detail in the Methods section. Detailed participant feedback about the paintings used, the activities carried out using the paintings, problems with using paintings and the role of paintings in future modules has not been previously carried out. Hence the present study was done to obtain detailed feedback about the use of paintings from the student participants.

## Methods

The present module for first year medical students was conducted from February to August 2010 at KIST Medical College (KISTMC), Lalitpur, Nepal (near Kathmandu). The 100 students admitted in the 2009 intake were divided into two batches of 50 students each. Students have early clinical exposure right from the first week of the MBBS course. MH sessions were held on alternate Thursdays during early clinical exposure for a particular batch of students from 8.30 to 9.45 am. As in previous modules case scenarios, role-plays, debates, paintings and other activities were used to explore different aspects of MH. Students were divided into five small groups with ten students in each. Each group was named after a well recognized scholar in MH. For example a group was named 'Coulehans' after Jack Coulehan, another 'Shapiros' after Johanna Shapiro, a third 'Acunas' after Leopoldo Acuna, all well recognized scholars in MH. The topics covered in the module were empathy, the patient, the doctor, the doctor-patient relationship and what it means to be sick in Nepal. Each topic except the last one was covered in two parts called 'bytes'. There was a brief presentation by the facilitators followed by different activities. 'Khula manch' (open space in Nepali) a forum for participants to express their views, that had been used in previous modules, was continued in this module also.

Table 1 shows the different paintings used in the module. The activities undertaken by the students included discussing 'What do you see' and 'What do you feel' about the painting, creating a story of 100 words about the scene depicted in the painting, creating a song/poem about the painting and interpreting the painting using role plays.

**Table 1:** List of paintings used in the module

Painting	Artist
'City hospital'	Alice Neel

'The paralytic'	Jean-Baptiste Grueze

'Portrait of Dr. Gachet'	Vincent Van Gogh

'Laennec Listening with his Ear Against the Chest of a Patient at the Necker Hospital'	Theobold Chartran

'Before the shot'	Norman Rockwell

'The dropsical woman'	Geritt Dou

'The doctor's visit'	Jan Steen

'The doctor'	Sir Lukes Fields

'The old guitarist'	Pablo Picasso

'The Agnew clinic'	Thomas Eakins

'The Anatomy Lesson of Nicolaes Tulp'	Rembrandt

'The quack'	Geritt Dou

'TB Harlem'	Alice Neel

'Tree of hope'	Frieda Kahlo

'Goya Attended by Dr. Arrieta'	Francisco Goya

'Gassed'	John Sargent

At the end of the module in August 2010 we obtained detailed feedback from the student participants about the paintings used in the module using a semi-structured questionnaire. The authors explained the objectives of the study to students who were then invited to participate. Written informed consent was obtained from all study participants. The study was approved by the Institutional Research Committee of KIST Medical College. The questionnaire used is shown in the Appendix. The questionnaire was tested for readability and ease of understanding among six second year students.

The demographic information collected was gender and method of financing of medical education. Information on the method of financing was obtained as self-financing students have to pay for their education and usually come from higher socioeconomic groups compared to scholarship students. Participants were asked to give two overall comments about the use of painting in 'Sparshanam', were asked how the paintings helped in realizing the module objectives; whether they were aware of the use of paintings in MH programs elsewhere; whether they have visited the Literature, Arts and Medicine database on the web; their level of enjoyment of the paintings; whether they felt the use of paintings from a western context was appropriate; their views of the painting annotations; which among the various paintings used could they identify with the most and the least. They were also asked to comment on their experiences with the different exercises used with the paintings (information was not collected regarding use of poems/songs and role-plays) and make possible suggestions regarding use of paintings in future modules. Two advantages according to the participants of using paintings and two suggestions to further improve the use of paintings were asked. The participant responses were collected and grouped under different headings and then either quoted verbatim or paraphrased by the authors.

Participants were asked to rate on a scale of 1 to 5 their enjoyment of the use of paintings in the module, their perception regarding the usefulness of annotations about the paintings and how useful they felt paintings in the module were. The median score was calculated for each statement and compared among male and female students using appropriate non-parametric tests (p < 0.05).

The comments for each question in the questionnaire were grouped together into similar headings and the number of times each comment or a comment similar to one already stated occurred was noted. Common comments (by six or more respondents) were noted using the author's own words or paraphrased by the authors as mentioned in appropriate places in the Results section.

## Results

*Basic demographics of participants: *Seventy-eight of the 100 students (78%) participated in the study. Thirty-four students (43.6%) were male while 44 were female. Seventy-two students (92.3%) were self-financing while six were scholarship students.

### Comments about the use of paintings in the module

The most common overall comments about the use of paintings in the module were: "they helped me feel what I saw" (12 respondents), "enjoyed the sessions" (12 respondents), "some paintings were hard to interpret" (10 respondents), "were in tune with the module objectives" (10 respondents), "gave information about history associated with medicine" (9 respondents) and "paintings improved the power of thinking" (8 respondents). Only thirteen students (16.7%) had been exposed to the use of paintings in school for educational purposes before. According to students paintings helped in realizing the objectives of the module in various ways. The paraphrased comments of 9 respondents were: paintings helped them develop feelings and visualize in a more concrete fashion the module objectives. Other comments were: "helped to see and feel things" (8 respondents), "portrayed different conditions" (6 respondents) and "helped see other points of view" (5 respondents).

### Use of Western paintings

Only two students (6.4%) were aware of the use of paintings in MH programs elsewhere. Forty-eight students (61.5%) felt the use of Western paintings was appropriate while 17 (21.8%) felt it was not entirely appropriate. Those who responded yes to this question felt dis-ease, empathy and other human feelings were common all over the world while those who said no felt Nepalese paintings may be more effective and there are cultural and other differences between Nepal and the west. Suggestions (paraphrased by the authors) to make annotations more useful were to make them shorter and more precise, make the language simpler and properly introduce the painter.

### Difficulties with paintings

Table 2 shows paintings with which participants could identify the most and cites reasons given. 'The portrait of Dr. Gachet' by van Gogh and 'The paralytic' by Jean-Baptiste Grueze were paintings with which they could identify the least. As regards the painting of Dr. Gachet reasons given were: "students could not understand what the scene was about, it was difficult to interpret and they were confused about who the person actually was".

**Table 2:** Paintings with which students identified the most

Painting	Number of respondents (percentage of total) N = 78	Reasons
City hospital	12 (15.4)	Eye catching, dark nature of painting, pain of the patient
Old guitarist	9 (11.5)	
The field	9 (11.5)	Crutch stands for disability, says so much, produced deep feelings
Tree of hope	7 (9)	Showing hope for a change, not gloomy Good story behind it
Anatomy lesson	6 (7.7)	

Forty-one students (52.6%) had difficulty with the exercise 'what do you feel' on seeing a particular painting. Thirty-one (39.7%) did not have any difficulty. Group work helped in resolving difficulties. Among the problems felt with this exercise were "difficulty in distinguishing feelings from what they saw, lack of vocabulary to express what they saw, some paintings were vague and they did not have previous exposure to the exercise".

### Activities connected to paintings

Table 3 shows the perception of participants regarding the exercise of creating a story of about 100 words about the scene depicted in the painting and their common suggestions to further improve the exercise. Fifty-two students (66.7%) had a certain amount of difficulty in putting the Western paintings in a Nepalese context. The difficulties were overcome by using ideas from all group members, correlating the painting with a Nepalese context, taking help from facilitators and trying to put themselves in the context represented by the paintings. Seventy-four students (94.9%) wanted paintings from Nepal to be included in the module. According to them paintings could be obtained from art exhibitions and help asked for from Nepalese artists. A useful suggestion was paintings and sketches by faculty and students could be used. Photographs could also be used in future modules. Students were strongly in favor of using paintings in future modules with 73 students (93.6%) saying yes. Table 4 shows the cited advantages of using paintings in medical education.

**Table 3:** Respondents' perception about the activity create a story of 100 words about the painting

Perception	Number of respondents (percentage) N = 78	Reasons (in decreasing order of frequency)
Useful	72 (92.3)	Helps in creatively exploring one's talents (14 respondents) Improves thinking & imagination (13 respondents) Helps to form own perspective regarding the scene (7 respondents) Nice way to express feelings (6 respondents) Interesting way of learning (6 respondents)

Not useful	3 (3.8)	

**Table 4:** Advantages of learning using paintings for medical students

Stated Advantage	Number of respondents (Percentage of total) N = 78
Promotes empathy & feelings	23 (29.5)
Creative & enjoyable activity	17 (21.8)
Increases thinking capacity	10 (12.8)
Provides opportunity to share ideas with group	7 (9)
Sensitizes students to future scenarios	7 (9)
Underlines importance of doctor-patient relationship	6 (7.7)
Improves power of imagination	6 (7.7)
Refreshing break from theory classes	6 (7.7)
Promotes lateral thinking	5 (6.4)

### Suggestions for further improvement and median scores

Suggestions given to further improve the use of paintings were: "use paintings showing the miserable condition of Nepalese people, use paintings by modern artists also, give more time to the exercise and use better picture quality for some of the paintings".

The median scores for the parameters: enjoyment of paintings, usefulness of annotations and usefulness of paintings was 4. Table 5 shows the median scores according to the gender of the respondents. There was no significant difference in scores between self-financing and scholarship students or between male and female respondents.

**Table 5:** Median and mean total scores of specific parameters according to gender of respondents

Parameter	Mean scores (± SD)			Median scores		
	**Male**	**Female**	**P value**	**Male**	**Female**	**P value**

Enjoyment of paintings	3.86 (1.28)	3.86 (1.28)	1	4	4	0.916

Usefulness of annotations	3.31 (1.28)	3.66 (1.15)	0.237	4	4	0.148

Usefulness of paintings	3.83 (1.13)	3.86 (1.33)	0.917	4	4	0.493

## Discussion

Overall student opinion about the paintings was positive. The paintings helped them feel what they saw and were generally enjoyable. Students identified better with certain specific paintings and had difficulty with the exercise 'what do you feel'. They also had problems extrapolating the context depicted in Western paintings to Nepal. They wanted paintings by Nepalese artists and if possible paintings and sketches by students and faculty to be used in the module.

### Interpretation of art in Nepal

Interpretation of art may be difficult for medical students in Nepal for a variety of reasons. In Nepal and South Asia students usually enter medical school after twelve years of schooling with the subjects of Physics, Chemistry and Biology in the last two years [14]. Drawing, arts and crafts and Humanities in general do not get the attention they deserve in school. Teaching methods and assessments stress factual recall of information. Also the top academic performers in school usually opt for the science stream and the 'arts and humanities' stream is looked down upon as students with a weaker academic performance usually opt for these. Also there is no popular culture of leisure reading and art appreciation.

### Paintings and the module objectives

The specific objectives of the module have been mentioned in the Introduction. We examined the connection between paintings and module objectives in the questionnaire in two ways, one directly by asking students how paintings helped in realizing module objectives and the other indirectly by asking the advantages of paintings for medical students. In the overall comments only 10 respondents explicitly felt the use of paintings was in tune with module objectives. We did not directly ask respondents whether the use of paintings was in tune with the module objectives. The respondents were only asked their overall comments and it may be that others had the same opinion about the module which they did not however put on paper.

Table 3 shows participants' perception regarding the activity of creating a story of 100 words. The activity improved thinking and imagination, helped in creatively exploring talents and in expressing feelings among others. These will be indirectly useful in advancing the module objectives. The effect of the module on empathy of participants is being studied. Imagination will be useful in putting oneself in the shoes of another person and indirectly improving empathy. More creative thinking may help in understanding the patient perspective of sickness and in developing a better doctor-patient relationship and being better able to express feelings can lead to a better doctor-patient relationship. Improved thinking, imagination and creativity can help to better understand the impact of sickness of a loved one on the family. Art has been used to improve empathy, emotional recognition, and identifying with and improving compassion towards the sufferer in the west [11,15-17]. In Table 4 the stated advantages of paintings were: promoted empathy and feelings, the activity was creative and enjoyable and increased thinking capacity. The opportunity to share ideas and work together in a group can lead to better team work which is important in today's practice. Sensitizing students to future scenarios will help in future practice. The session being considered interesting and being a refreshing break from theory classes may lead to better attendance in and participation in module activities. Many of the advantages stated were indirectly linked to the module objectives as stated previously.

The questionnaire focused on many aspects of use of paintings in the module and linkage with module objectives was only as a small but important portion. Formative student feedback is taken at the end of each session concentrating on whether the various learning modalities used were relevant to session objectives. Overall feedback suggests students felt session objectives were met. However, we have to reassess whether paintings were effective in meeting the overall module objectives as only few respondents stated this explicitly during the study. Also this was participants' perception and students who had undergone the module should be followed during the clinical years of study to observe various desirable characteristics. We do not have a control group for comparison as we are a new medical school and all student batches have undergone the module.

### Paintings from Nepalese artists

Respondents wanted paintings from Nepal and by Nepalese artists. We are working on modalities of collaborating with Nepalese artists and art galleries. With artists permission photographs of their paintings with annotations can be prepared. Finance is a major constraint as the program is not supported by any external agency and has no specific funds earmarked for it in the institution. We are also working on organizing a competition of sketches and paintings for faculty and students on specific health related themes. The paintings and sketches can be used in future modules. Certain respondents suggested shorter, simpler and more specific annotations.

### Scores according to gender and method of financing of education

There were no significant differences in scores between male and female students and between scholarship and self-financing students. The number of scholarship students was low and only 6 of the 10 students participated in the study.

### Study limitations

The study had limitations. It was carried out in a single institution in Nepal. The batch of 100 students in our institution has 51 males and 49 females. The participation of males in the study was low. Feedback was taken during the last MH session and by then students may have been concentrating on their assessments. Not all students participated in the survey. It could be possible that the students who participated were more favorably disposed towards the use of paintings in medical education. The study was semi structured with the points to be discussed mentioned in the questionnaire and respondents were free to answer according to their wishes. This may have created diversity of responses which were difficult to group together under specific themes. Also paintings were only one of the many modalities used in the module. Detailed analysis of particular responses using methods like semi-structured interviews or focus group discussion was not carried out.

### Potential clinical implications of study findings

Helping students feel what they see may have implications in clinical practice. A major problem is that patients complain that doctors do not have feelings and often do not consider them as human beings (i.e., as equals). In a previous study conducted at Manipal College of Medical Sciences, Pokhara it was noted that students who completed the voluntary module developed a more comprehensive approach towards patients and considered the patient in a holistic setting [7]. An identification with and empathy towards patients may help in creating more compassionate doctors. Creative thinking or thinking out of the box will be useful at a community level and beyond in finding solutions to Nepal's pressing health problems. At an individual patient level it may help in finding new solutions to patient problems which have not benefited from repeated medical consultations.

## Conclusions

The study shows that it is possible to use paintings in medical education in a developing country. Participant response was positive. There were a number of advantages of using paintings. The arts can free the imagination, help students see new perspectives and think critically and creatively about problems they may encounter in their future career. Students had occasional problems in applying the western scenario depicted in the paintings to Nepal. Use of more paintings from Nepal can be considered. Paintings can be an interesting method of teaching students. Student satisfaction with the use of paintings in the module was high. Paintings and MH modules can be considered for inclusion in other medical schools.

## Competing interests

The authors declare that they have no competing interests.

## Authors' contributions

PRS conceptualized the study, designed the questionnaire, analyzed the questionnaire, interpreted the results and wrote the paper. RMP helped in conceptualizing the study, designing and administering the questionnaire, conducting the sessions, analyzing results and writing the paper. KUD helped in study conceptualization, designing and administering the questionnaire, reviewing literature and writing the paper.

All were involved as facilitators in the Medical Humanities module.

## Appendix: Questionnaire used in the study

Student feedback about the use of paintings in Sparshanam, the KISTMC Medical Humanities module

Gender: M/F Self-financing/Scholarship

Give TWO overall comments about the use of paintings in Sparshanam:

Have you exposed to the use of paintings for educational objectives before? If yes, give details.

According to you how did paintings help in realizing the objectives of the module? (Two points)

Are you aware of the use of paintings in medical education elsewhere? If yes, give mention them.

Do you enjoy the use of paintings in the module? Grade on a scale of 1 to 5 with 1 being least and 5 being most enjoyable.

Do you feel use of paintings from a western context were appropriate in the module?

Give your reasons.

Were the annotations presented about the paintings useful?

Grade on a scale of 1 to 5 with 1 being least and 5 being most useful.

Give TWO suggestions to make use of annotations more useful.

Which of the various paintings used could you identify with the most? Why?

Which of the various paintings used could you identify with the least? Why?

Do you feel you had difficulty with the exercise 'What do you feel'? Why?

Do you feel the exercise write a story of 100 words about the scene depicted in the painting is useful? Give your reasons

What would you suggest to make this exercise more useful?

Did you have any difficulty in putting the western scenarios depicted in paintings in a Nepalese context?

If yes, then how did you overcome these?

Do you feel paintings from Nepal should be used in the module?

If yes, then how should we go ahead?

Do you feel paintings should be used in future modules?

Mention TWO advantages of paintings for medical students.

How would you rate the use of paintings in the module on a scale of 1 to 5 (with 1 being least and 5 being most useful)

Give TWO suggestions to further improve the use of paintings in future

Thank you for completing the questionnaire. It is very much appreciated!

## Pre-publication history

The pre-publication history for this paper can be accessed here:

http://www.biomedcentral.com/1472-6920/11/9/prepub
